# Reevaluating the Design of Multicenter Surgical Trials for Esophagogastric Cancer

**DOI:** 10.1245/s10434-025-18532-x

**Published:** 2025-10-23

**Authors:** Bibek Das, Anuja T. Mitra, Patrick Bossuyt, George B. Hanna

**Affiliations:** 1 Division of Surgery, Department of Surgery and Cancer, Imperial College London, London, UK; 2 Department of Epidemiology and Data Science, Amsterdam Public Health, Amsterdam UMC, University of Amsterdam, Amsterdam, The Netherlands

**Keywords:** Surgical oncology, Randomized controlled trials, Surgical quality, Trial methodology

## Abstract

**Background:**

Multicenter randomized controlled trials (RCTs) in esophagogastric cancer surgery have repeatedly failed to demonstrate overall survival (OS) benefits for technical interventions. This raises concerns about trial design frameworks, particularly surgical quality assurance (SQA) and their impact on statistical power and sample size requirements.

**Methods:**

We conducted a systematic review of MEDLINE, Embase, and Cochrane CENTRAL (January 1990 to June 2024) to identify multicenter RCTs evaluating curative surgical interventions for esophageal or gastric cancer that reported OS outcomes with ≥ 12 months of follow-up. Trials were assessed for SQA strategies, protocol adherence, and sample size assumptions. Post hoc power analyses and statistical simulations modeled the impact of surgical variability on trial outcomes.

**Results:**

We identified 27 eligible RCTs; 10 were powered for OS superiority, but none showed significant OS benefit. Suboptimal SQA was common, including limited surgeon credentialing, poor adherence monitoring, and inadequate technical standardization. Three trials were underpowered at design, and three failed to meet recruitment targets. Simulations showed that a technical nonadherence rate of 10% per arm could halve statistical power. Sample size requirements to preserve 80% power increased markedly, sometimes by thousands of participants. Most trials lacked internal piloting to assess protocol feasibility and baseline adherence.

**Conclusions:**

Inadequate SQA and failure to account for surgical variability may partly explain the lack of OS benefit in multicenter surgical RCTs. Embedding SQA and internal pilot studies into trial design may improve feasibility, ensure protocol fidelity, and strengthen the validity of future trials in complex oncologic surgery.

**Supplementary Information:**

The online version contains supplementary material available at 10.1245/s10434-025-18532-x.

Cancers of the esophagus and stomach represent a significant global healthcare challenge and are associated with high mortality. In 2022, there were 1,479,066 new cases of esophagogastric cancer and 1,104,982 associated deaths globally.^1^ This highlights an important need to identify treatment strategies that could improve patient survival.

The cornerstone of curative treatment for esophagogastric cancer is en bloc resection of the tumor and involved lymph nodes with negative margins. In recent decades, there have been a number of landmark practice-changing trials in esophagogastric cancer, such as MAGIC,^2^ CROSS,^3^ and FLOT,^4^ that have demonstrated an improvement in overall survival (OS) with a multimodal strategy that combines systemic treatment with radical surgery. Compared with chemotherapy trials, however, surgical RCTs have not demonstrated similar convincing survival benefits. These negative results have led to a wide variation in practice with uncertain implications for patient outcomes.

While multicenter RCTs represent the highest level of evidence, relying on these trials in surgery carries a risk of increasing heterogeneity in the effect of treatment, due to variation in individual technical performance. This is particularly relevant for esophagogastric cancer surgery, which is complex and where trials have often evaluated an isolated technical modification. Surgical quality assurance (SQA) measures to ensure standardization of technique—both the individual technique under evaluation and the procedure overall—are essential within a multicenter trial to ensure sufficient uniformity in both trial arms. A review of esophagogastric cancer trials has previously shown that credentialing surgeons by evaluating procedural volume and operative reports, along with the standardization of surgical techniques, led to a decrease in in-hospital mortality rates.^5^ Additionally, assessing operative reports and monitoring performance data contributed to reduced variability in operative techniques within the trial, as indicated by the consistency in lymph node harvest.^5^ Nonetheless, adherence to protocolized technique remains variable and is often unmeasured, particularly in multicenter settings.

Unmeasured protocol deviations and suboptimal SQA could substantially reduce statistical power and limit the estimated effect size of an intervention, placing trials at high risk of false-negative outcomes. We aimed to review data from multicenter RCTs of curative esophagogastric cancer surgery with a focus on SQA, to identify methodological factors that could bias outcomes leading to negative results and to explore new paradigms in surgical trial design.

## Methods

### Search Strategy and Selection Criteria

A systematic review of the literature was performed according to Preferred Reporting Items for Systematic Reviews and Meta-Analysis (PRISMA) guidelines (checklist in Supplementary Table 1).^6^ The aim was to identify articles reporting esophageal and gastric cancer surgical RCTs with survival outcomes published between 1 January 1990 and 13 June 2024. Full search strategies for the MEDLINE, Embase, and Cochrane CENTRAL databases are outlined in Supplementary Table 2. Reference lists of selected articles were screened to identify additional potentially relevant articles. The search was limited to articles published in the English language where the full text was available. Study protocols, conference abstracts, and review articles were excluded. The search results were imported into the Covidence platform, and duplicate removal was performed.

Two reviewers (B.D. and A.M.) independently screened abstracts and performed full-text reviews of potentially relevant articles to decide on eligibility for inclusion. Any disagreements were resolved by a third reviewer (G.B.H.). Studies were included if they met the following criteria: (i) the study was a multicenter RCT; (ii) the focus of the RCT was a technical aspect of esophageal and/or gastric cancer surgery (e.g. extent of resection, surgical approach, or lymphadenectomy); (iii) surgery was performed with curative intent; (iv) OS was a reported outcome; (v) the reported OS data was ≥ 12 months. Nontechnical intraoperative adjuncts such as peritoneal lavage were excluded. OS is the preferred endpoint in oncological trials as the primary goal of cancer treatment is to extend survival.^7^

### Outcomes

The following baseline data were extracted from the final selection of articles: author, year of publication, recruitment period, base country, number of centers, study focus, type of cancer, and numbers of participants in each arm including power calculation parameters (power, alpha, expected difference, accrual time, attrition rate, planned sample size, and actual sample size). Trials were then grouped into three categories depending on the primary outcome and power calculation: group 1, powered for overall survival (superiority); group 2, powered for overall survival (noninferiority); group 3, not powered for overall survival (i.e., disease-free survival or other nonsurvival endpoint). Group 1 RCTs were the focus of this evaluation as these trials were designed to demonstrate whether the technique under investigation could improve overall survival compared with the control. Overall survival data (percentage and univariate hazard ratio) from the intention-to-treat analysis were extracted from each trial.

### SQA Assessment

An assessment of SQA for each trial was evaluated using a standardized framework^5^ by two independent reviewers (A.M. and B.D.). The elements of the ten-point checklist included trial design factors deemed critical for SQA: pretrial surgeon education; surgical approach standardization; standardization of the extent of lymphadenectomy; case volume; operative reports’ videos to credential surgeons; live operating room evaluation; videos to monitor operative performance; data monitoring by an independent committee; centralized pathology review. Risk of bias was also assessed using the Cochrane method.^8^

### Statistics

Univariate hazard ratios or calculated OS differences between control and intervention groups (with confidence intervals estimated using the delta method^9^) were used to express the estimated effect size of surgical interventions. Hazard ratios were estimated using the methods described by Parmar^10^ and Tierney^11^ when not explicitly reported. A forest plot and heterogeneity estimate with a random effects model were generated using the *meta* package in R. Post hoc study power was estimated using the *cpower* function of the *Hmisc* package and compared with the stated power. This method assumes exponential survival distributions for both treatment groups. Calculations of study power were made using the same assumptions as stated in study protocols. The following parameters were extracted from the study protocols and used to estimate the power using *cpower*: total sample size excluding attrition, time at which mortalities were estimated, number of subjects in control and intervention groups, type I error probability, duration of accrual period, and minimum follow-up time.

For studies that did not meet the planned sample size, estimated sample sizes for follow-up studies were calculated using the *nsurvival* function of the *gsDesign* package. A fixed design was used, applying the method recommended by Lachin and Foulkes^12^. The power was set at 80% with a two-sided alpha 0.05. Study maximum duration was the primary outcome duration (e.g., 5 year OS), and accrual time was 2/3 maximum duration. Dropout rate was set at 0%, and a 1:1 split ratio was used for control versus intervention.

Study power as a function of technical nonadherence in the JCOG1001 trial was simulated using the *cpower* function of the *Hmisc* package. The original trial assumptions were used: 25% mortality in no bursectomy versus 20% mortality in bursectomy group, implying a 20% reduction in mortality with bursectomy, and alpha of 0.05. Estimates of power were made with technical nonadherence ranging between 0 and 25% in control and intervention groups. The point sample size requirement versus mortality reduction assuming a constant power of 80% was estimated using a root finder function (*uniroot*) from the *stats* package. All statistical analyses were performed using R version 4.4.1.

## Results

**Fig. 1.**
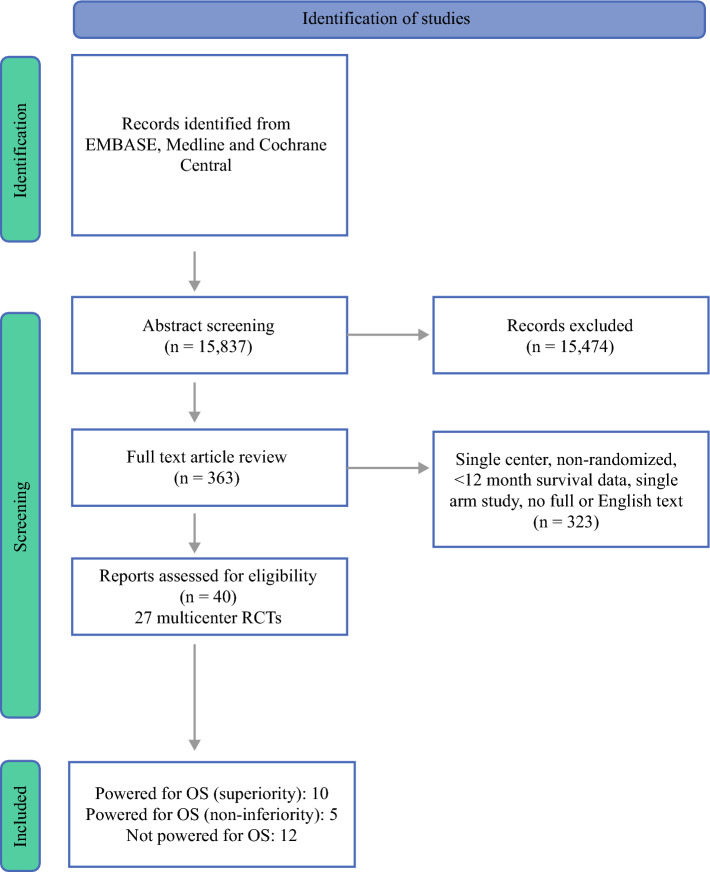
PRISMA workflow

An initial search identified 15,837 articles, of which 15,474 were deemed not eligible following title and abstract screening. Full-text assessment was performed for 363 articles, of which 40 were included. These reported on 27 multicenter RCTs, as several RCTs had more than one publication reporting outcome data at different time intervals. Of these RCTs, 10 were powered for overall survival (superiority, group 1), 5 were powered for overall survival (noninferiority, group 2), and 12 were not powered for overall survival (group 3). The PRISMA search workflow is shown in Fig. 1.

The study characteristics of group 1 RCTs are displayed in Table 1, and those of group 2/3 RCTs in Supplementary Table 3. Among group 1 RCTs, seven focused on treatment of gastric cancer, two on the treatment of esophageal cancer, and one included both esophagogastric junctional and gastric cancers. Risk of bias for group 1 RCTs was low, aside from a lack of blinding in eight out of ten RCTs (Supplementary Table 4).

**Table 1.** Characteristics and survival outcomes of multicenter surgical trials for esophagogastric cancer powered for overall survival

Trial	Author, year	Recruitment	Base country	Site	Study focus (A versus B)	*N* (A versus B)	Centres	OS duration	OS (A) (%)	OS (B) (%)	Univariate HR (A/B)	*P* value
Dutch D1D2	Bonenkamp 1999^23^	1989–1993	Netherlands	Gastric	D2 versus D1 gastrectomy	331 versus 380	80	5-year survival	33 ± 2.2	34 ± 2.1	1.09 (0.94–1.27)	—
	Hartgrink 2004^24^							11-year survival	35	30		0.53
	Songun 2010^25^							15-year survival	29.0 (24.0–34.0)	21.0 (17.0–26.0)		0.34
Dutch THvsTT	Hulscher 2002^26^	1994–2000	Netherlands	Esophageal	Transthoracic versus transhiatal esophagectomy	114 versus 106	2	5-year survival	39.0 (30.0–48.0)	29.0 (20.0–38.0)	0.97 (0.83–1.14)*	0.38
	Omloo 2007^27^							5-year survival	36	34		0.71
FASR	Gouzi 1989^18^	1980–1985	France	Gastric	Subtotal versus total gastrectomy	76 versus 93	Multiple	5-year survival	48	48	1.00 (0.66–1.52)*	—
	Gouzi 1994^19^							5-year survival	48	48		—
IGCSG D1D2	Degiuli 2014^28^	1998–2006	Italy	Gastric	D2 versus D1 gastrectomy	134 versus 133	5	5-year survival	64.2	66.5	1.18 (0.85–1.65)	0.695
	Degiuli 2021^17^							15-year survival	46.8 (39.0–56.0)	51.3 (43.0–61.0)		0.314
Japan D2+PAND	Sasako 2008^29^	1995–2001	Japan	Gastric	D2+PAND versus D2 gastrectomy	260 versus 263	24	5-year survival	70.3 (64.3–75.4)	69.2 (63.2–74.4)	1.03 (0.77–1.37)	0.85
Japan D2D4	Yonemura 2008^30^	1995–2002	Japan	Gastric	D4 versus D2 gastrectomy	134 versus 135	10	5-year survival	55	52.6	0.93 (0.53–1.63)*	0.801
JCOG1001	Kurokawa 2018^21^	2010–2015	Japan	Gastric	Bursectomy versus no bursectomy	602 versus 602	57	5-year survival	76.9 (72.6–80.7)	76.6 (72.0–80.6)		—
	Kurokawa 2023^31^							5-year survival	74.9 (71.2–78.2)	76.5 (72.8–79.7)	1.03 (0.83–1.27)	0.81
JCOG9502	Sasako 2006^16^	1995–2003	Japan	GEJ/gastric	LTA versus transhiatal esophagectomy	85 versus 82	27	5-year survival	37.9 (26.1–49.6)	52.3 (40.4–64.1)	1.36 (0.89–2.08)	0.92
	Kurokawa 2015^15^							10-year survival	24.0 (15.0–34.0)	37.0 (26.0–47.0)	1.42 (0.98–2.05)	0.06
MRC ST01	Cuschieri 1996^32^	1990–1995	UK	Gastric	D2 versus D1 gastrectomy	200 versus 200	5	5-year survival	30	50		0.05
	Cuschieri 1999^33^	1990–1997	UK	Gastric	D2 versus D1 gastrectomy	200 versus 200	5	5-year survival	33	35	1.10 (0.87–1.39)	0.43
NST1501	Mao 2022^34^	2015–2018	China	Esophageal	Left versus right thoracic esophagectomy	453 versus 408	14	3-year survival	68.7	71.3	0.85 (0.65–1.13)	0.2

^*^Estimated hazard ratios

*GEJ* gastroesophageal, *HR* hazard ratio, *LTA* left thoracoabdominal, *OS* overall survival, *PAND* para-aortic lymph node dissection

**Fig. 2.**
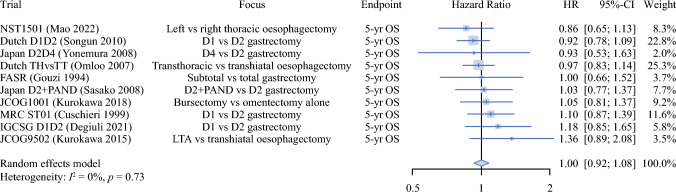
Forest plot of hazard ratios from group 1 multicenter surgical trials for esophagogastric cancer

The primary survival outcomes in these trials are listed in Table 1. Hazard ratios for group 1 trials are shown in a forest plot in Fig. 2, and between-study heterogeneity was low (*I*^*2*^ = 0%, *p* = 0.73). We observed that no multicenter RCT designed to show superiority of a surgical technique for esophagogastric cancer had shown a statistically significant survival benefit. This was equally the case for studies where demonstrating an improvement in OS was not the primary focus of the trial (Supplementary Table 3). Estimated effect sizes (OS difference and hazard ratios) from these trials were low and not statistically significant (Supplementary Table 5). Of note, all group 2 RCTs confirmed noninferiority of their interventions except for the Osaka bursectomy trial.^13,14^

Next, we sought to explore potential study design factors that could account for these negative results. The assessment of surgical design and quality of group 1 RCTs is summarized in Supplementary Table 6. The majority of RCTs included methods to standardize surgical techniques, using pretrial education (6/10), standardization of the surgical approach (8/10), and standardization of the extent of lymphadenectomy (10/10). However, only a minority of studies included methods to credential surgeons, such as setting a minimum case volume (5/10), reviewing operative reports (1/10), video assessment (1/10), or live operating room assessment (2/10). Furthermore, a minority of studies included methods to monitor performance such as video submission and assessment (5/10) or centralized assessment of pathology (2/10).

The details of sample size calculations used for group 1 RCTs are summarized in Supplementary Tables 7 and 8. This showed that, in 3/10 trials, the calculated study power based on these assumptions was less than 80%, ranging from 57 to 72%. Furthermore, in 3/10 trials, the planned sample size was not achieved. For JCOG9502, recruitment was stopped early as an interim analysis showed that patients assigned to the intervention under investigation (left thoracoabdominal approach) were unlikely to have improved overall survival compared with the transhiatal approach.^15,16^ For IGCSG-R01, the authors noted that the trial had lower than expected recruitment.^17^ Similarly, in the FASR gastrectomy trial, 32/201 (15.9%) recruited patients were excluded after trial completion, which led to a lower-than-expected number of patients in the final analysis.^18,19^ Sample size calculations were repeated for these trials, to estimate the number of patients required to demonstrate a statistically significant improvement in survival in a follow-up study (Supplementary Table 9). This revealed that large numbers of patients, ranging to the thousands, would be required given the small effect sizes in these studies.

**Fig. 3.**
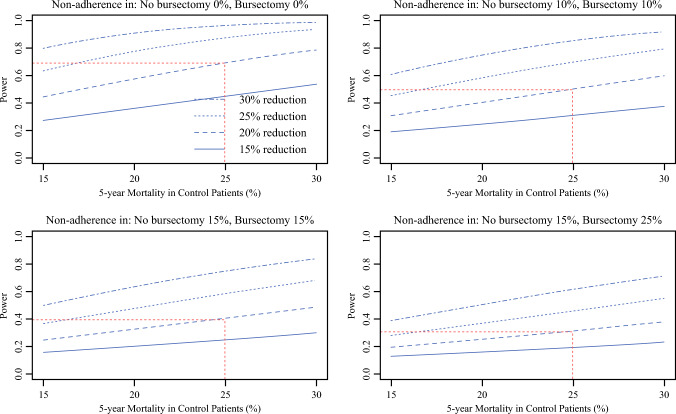
Simulations of protocol nonadherence in JCOG1001 and impact on study power

Finally, given that the assessment of trial design revealed potential sources of technical variance, we simulated the impact of unmeasured technical variance (protocol nonadherence) in the JCOG1001 bursectomy trial. This revealed a significant reduction in study power with increasing protocol nonadherence (Fig. 3). For example, a 10% rate of nonadherence in each trial arm would lead to study power falling to ~50%. Simulated sample size requirements with fixed nonadherence rates revealed that thousands of patients would be required to demonstrate 15–25% mortality reduction with 80% power (Supplementary Fig. 1).

## Discussion

This review showed that no multicenter surgical RCT for esophagogastric cancer had demonstrated an overall survival benefit that was statistically significant. We reviewed study design factors that may have contributed to these negative results. Some trials did not meet their planned sample size owing to low recruitment and attrition, and some trials may have been underpowered from the onset. Importantly, we observed a substantial degree of heterogeneity in SQA, such as surgeon credentialing before enrollment or monitoring performance during the trials. We demonstrated that even a low degree of protocol deviations that are not accounted for during study design could lead to trials being considerably underpowered.

Much has been written on the importance of SQA during multicenter trials to limit technical variability as a source of bias. Few trials, however, have reported rates of technical nonadherence to study protocols during trials, although there is evidence that this is a significant issue. For example, in the Dutch D1 versus D2 gastrectomy trial, a post-trial analysis revealed that 52% of operations in the D1 resection group had more widespread dissection than specified, and 84% of operations in the D2 gastrectomy group had less dissection than specified.^20^ This degree of heterogeneity is likely to significantly limit the power of the trial and the estimated treatment effect if the rates of technical nonadherence are not factored into the study design. Addressing quality control factors such as video assessment and standardized pathological assessment will be essential in the design of future surgical trials to ensure homogeneity. Additionally, our analysis highlights the value of measuring the rate of baseline protocol nonadherence in a pilot study after the implementation of SQA to improve the accuracy of sample size calculations.

Another important question is whether it is pragmatic to evaluate a single component of a complex operation in a multicenter RCT setting, owing to variation in technique between individual surgeons taking part in RCTs, the large numbers of patients that would be required, and unmeasured confounding variables (e.g., tumor biology). Our sample size calculation simulation showed that thousands of patients would be required to demonstrate a mortality benefit from bursectomy with relatively low rates of protocol nonadherence. Multicenter RCTs are expensive and require significant resources to deliver. Two RCTs did not meet their planned sample size owing to attrition, which also highlights difficulty in patient recruitment. Altogether, this highlights the formidable challenges of delivering multicenter surgical RCTs in their current form.

Multicenter enrichment RCTs, recruiting patients most likely to respond to an intervention, may be an area of future exploration, similar to biomarker-stratified pharmaceutical trials. This could also significantly reduce the sample size required to show a survival benefit of an intervention. While post hoc analyses are susceptible to biases and false positives, such analyses have suggested that specific subgroups may be more likely to benefit from a technical intervention. For example, a survival benefit was shown for serosa-positive tumors from bursectomy.^21^ If there are only certain subgroups who substantially benefit, trials in allcomers are likely to have limited power. Methods for accurately selecting patients for inclusion into clinical trials on the basis of baseline characteristics that would favor a particular intervention are greatly needed.

A key strength of this review is the systematic appraisal of trial design and quality, which may have broader implications for surgical trials in general. There are also limitations of this review to consider. Although this appraisal has focused on study design and SQA, there may be other factors (e.g., age, tumor biology, and medical comorbidities) that could affect clinical outcomes, especially if randomization was incomplete. There are many methods of estimating study power and sample size. The methods chosen in this evaluation may have differed from the trial authors, although efforts were made to use the same underlying assumptions. Finally, surgery performed within the context of a multicenter RCT may not entirely reflect “real-world” practice owing to observer and operator bias. Hence, it is important not to discount results from registry data or data from large regional single centres^22^ to inform surgical practice.

In summary, multicenter RCTs in esophagogastric cancer surgery evaluating survival outcomes have not demonstrated statistically significant benefits. We identified trial features that may have contributed to these results, highlighting the complexities of conducting surgical RCTs in this setting. These findings suggest that refinements in trial methodology may be needed to enhance the validity, feasibility, and impact of future studies. Frameworks for assessing bias in RCTs have been developed primarily for pharmacological trials and may not fully account for the variability inherent in surgical interventions. Future surgical RCTs may benefit from enhanced SQA, real-world pilot studies to assess adherence to standardized protocols with more accurate sample size calculations, and improved patient stratification. A more tailored approach to trial design, incorporating these elements, could optimize the ability to detect meaningful clinical benefits while maintaining feasibility in multicenter settings.

## Supplementary Information

Below is the link to the electronic supplementary material.

Supplementary file1 (DOCX 84 KB)

Supplementary file2 (XLSX 30 KB)

## Data Availability

The authors confirm that the data supporting the findings of this study are available within the article and its supplementary materials.
