# Supplementary material for: Reevaluating the Design of Multicenter Surgical Trials for Esophagogastric Cancer
Source: Ann Surg Oncol. 2025 Oct 23;33(2):1007–15. doi: 10.1245/s10434-025-18532-x (PMC12765741; doi:10.1245/s10434-025-18532-x)
Supplement: Supplementary file 1 — Supplementary file1 (DOCX 84 KB) [file 10434_2025_18532_MOESM1_ESM.docx]

Supplementary Figure 1. Estimated sample size requirements in JCOG1001 with moderate protocol nonadherence at different mortality reduction thresholds
